# Microbial Inoculum Composition and Pre-weaned Dairy Calf Age Alter the Developing Rumen Microbial Environment

**DOI:** 10.3389/fmicb.2019.01651

**Published:** 2019-07-23

**Authors:** Laura M. Cersosimo, Wendy Radloff, Geoffrey I. Zanton

**Affiliations:** ^1^Department of Animal Sciences, University of Florida, Gainesville, FL, United States; ^2^United States Department of Agriculture (USDA)-Agricultural Research Service, Dairy Forage Research Center, Madison, WI, United States

**Keywords:** bacteria, protozoa, ciliates, microbiota, ruminant

## Abstract

The objective of this experiment was to determine if dosing pre-weaned calves with enriched ruminal microbiota alters the rumen microbial environment and growth performance. Twenty Holstein bull calves were removed from their dam at birth, fed 3.8 L colostrum within 4 h after birth, and housed individually. Calves were fed pasteurized milk 3×/d from 0 to 7 weeks of age and offered a texturized calf starter *ad libitum* at 6 days of age. A randomized complete block design with repeated measures and a 2 × 2 factorial arrangement of treatments was used to evaluate responses. Treatments were administered by stomach intubation once per week from 3 to 6 weeks of age and included: 50 mL autoclaved rumen fluid (RF), 50 mL bacterial-enriched RF (BE), 50 mL protozoal-enriched RF (PE); or 50 mL of each BE and PE inoculum. A rumen content composite was collected from 4 rumen fistulated, lactating cows and used to create the inocula. BE inocula were microscopically confirmed to be free of ciliate protozoa before inoculation, while PE contained 2.9 ± 2.2 × 10^5^ protozoa/mL. RF was collected from the calves once per week before 50 mL of the inoculum was administered. Animal performance (e.g., weight gain and dry matter intake) was not altered by inocula type. All calves were microscopically free of rumen ciliates before inoculum administration and calves that did not receive PE remained ciliate-free. Ciliate protozoa were observed in RF from 6, 8, and 6 PE treated calves (*n* = 10) at weeks 4, 5, and 6, respectively. Ruminal NH_3_ was lower in PE treated calves (3.3 vs. 6.8 ± 1.0 mM), while ruminal butyrate molar percent was greater in BE treated calves (10.8 vs. 8.3 ± 0.8). Rumen bacterial diversity measures did not differ by treatment at 3–6 weeks. Individual calf bacterial communities from treated calves became temporarily similar to the inocula at 4 weeks of age, but these communities diverged from the inocula at 5 weeks. This study provides new information about two types of rumen-derived inocula and insight into the challenges of directing the rumen microbial environment in the pre-weaned calf.

## Introduction

Dairy cattle require a diverse community of ruminal microorganisms (bacteria, fungi, protozoa) to convert plant polysaccharides to the energy substrates for growth and milk production. The rumen of a newborn dairy calf is non-functional and sterile (Baldwin et al., 2004) and increases in capacity from 30 to 70% of the total gastrointestinal tract (Warner et al., 1956; Castro et al., 2016). Cellulolytic bacteria and fungi (i.e., fiber fermenters) and archaea (i.e., methane producers) are the first microbiota to be present in the rumen (Fonty et al., 1987), whereas ciliate protozoa do not appear until later (Eadie, 1962a). The rumen microbial community continues to change as calves are transitioned from milk to solid feed (e.g., starter pellets and forage) with eventual establishment of a mature ruminal microbial community (Meale et al., 2016; Dill-McFarland et al., 2017). While rumen bacteria become established early, ciliate protozoa can be excluded indefinitely by isolating protozoa-free ruminants from faunated animals. Contact with older animals, aerosolized droplets, and calf management practices (Eadie, 1962b; Fonty et al., 1988; Williams and Coleman, 1992) are potential natural sources of ciliate protozoa inoculation into a young ruminant. Ciliate protozoa affect rumen fermentation through their capacity to ferment fiber into energy, predate bacteria as their main protein source, and accumulate starch and soluble carbohydrates (Williams and Coleman, 1992; Newbold et al., 2015).

Research efforts were made during the last decade to enhance productive performance of dairy cows through manipulation of their rumen microbial ecosystem (Yáñez-Ruiz et al., 2015). Several studies have demonstrated host-specificity and resiliency of ruminal microbiota from lactating cows (Jewell et al., 2015; Weimer, 2015; Weimer et al., 2017), suggesting that the established adult rumen ecosystem is resistant to perturbations (Malmuthuge and Guan, 2017). Near-total exchange of ruminal contents between high- and low-efficient cows resulted in the reestablishment of their pre-exchange ruminal bacterial communities within 10 days, while ruminal volatile fatty acid molar proportions and pH returned to pre-exchange values within 1 day (Weimer et al., 2017).

The developing rumen of the dairy calf provides a potential opportune period to direct microbial establishment (Yáñez-Ruiz et al., 2015). Early dietary interventions, probiotics, and adult rumen contents have been used in an attempt to modify the rumen and fecal microbial communities of young ruminants (Schönhusen et al., 2003; Ishaq et al., 2015; Fouladgar et al., 2016; Zhang et al., 2017). Rumen contents inoculated into calves have included the use of mixed ruminal microbiota from either whole rumen fluid or a ruminal bolus. Studies from the mid-1900s used either ruminal boluses or fluid from adults to inoculate calves to better understand early rumen microbial establishment (Pounden and Hibbs, 1950; Bryant and Small, 1960). In more recent studies adult rumen fluid was orally administered to inoculate and successfully faunate Holstein pre-weaned calves with ciliate protozoa at 5 and 6 weeks of age (Schönhusen et al., 2003) and Brahman heifers at 8 months of age (Nguyen and Hegarty, 2016).

Although previous inoculation studies have used whole rumen fluid or boluses to inoculate dairy calves, we are unaware of a study that has determined the effect of microbial composition type on the rumen microbial ecosystem and growth performance. Rumen fluid collected from an adult cow can be processed to remove ciliate protozoa by differential centrifugation or enriched with live rumen ciliates by gravimetric separation (Or-Rashid et al., 2007). As rumen fluid contains a diverse group of microorganisms with different functions, it is important to learn the potential impact of specific microbiota on the developing rumen. We hypothesized that microbial inoculum composition (bacterial- or protozoal-enriched) influences calf rumen microbial environment, thus altering ruminal fermentation end-products and calf growth performance. The objective of the experiment was to determine if the rumen microbial inoculum type affects the rumen microbial ecosystem of pre-weaned calves, thus influencing calf health and performance.

## Materials and Methods

### Experimental Design and Calf Management

The University of Wisconsin’s Institutional Animal Care and Use Committee approved all animal procedures under the protocol A005829. Holstein bull calves at birth (*n* = 20) were randomly assigned to a 2 × 2 factorial arrangement of treatments over a 4-week period between July 2017–August 2017. The four treatments (*n* = 5 each) included autoclaved, clarified rumen fluid (RF), bacterial-enriched inoculum (BE), protozoal-enriched inoculum (PE) (*n* = 5), or both BE and PE inocula. Calves were immediately removed from their dam at birth and housed separately from adult animals in individual calf hutches with sand bedding at the US Dairy Forage Research Farm in Prairie du Sac, WI. Average monthly temperatures and relative humidity, were: July (22.8 ± 3.6°C, 72.3 ± 10.8%), August (20.9 ± 3.5°C, 71.8 ± 9.7%), and September (19.8 ± 5.4°C, 69.0 ± 10.4%). At birth (d1), calves weighed 41.1 ± 5.6 kg and received colostrum with an average Brix score of 23.4 ± 2.7%. Calves were fed 2.5 L of pasteurized waste, antibiotic-free milk (2.9% protein, 2.9% fat, 2.7 × 10^2^ colony forming units) 3×/d from 2 days to 7 weeks of age, and offered Vita Plus BSF 18 calf starter (Vita Plus Corp., Madison, WI, United States) for *ad libitum* consumption at 6 days of age (Table 1). Calf starter was comprised of shell corn, soybean meal, cottonseed hulls, kibbled corn, cane molasses, and heat processed soybeans. As-fed starter and refusals were measured on a daily basis. According to the manufacturer’s analysis, calf starter contained monensin (40 g/ton) to prevent coccidiosis. Calf body measurements (bodyweight, body length, paunch and cardiac girths, wither and hip heights, hip width) were measured weekly, from 1 to 7 weeks of age. Weekly bodyweights were used to calculate average daily gain. Calf health assessments using the University of Wisconsin School of Veterinary Medicine’s Scoring Chart^1^ were performed twice a week from 1 to 7 weeks in age. Rectal temperatures, nasal discharge, eye, ear, and feces were scored from 0 to 3 with a score of 0 as normal.

**TABLE 1:** Nutrient composition of calf starter and pasteurized milk.

**Component**	**% dry matter basis**
**Calf starter**	
Dry matter (% as-fed)	91.3 ± 0.99
Crude protein	19.9 ± 0.81
Neutral detergent fiber	19.5 ± 3.94
Non-fiber carbohydrates	50.9 ± 3.68
Water-soluble carbohydrates	9.91 ± 0.49
Starch	38.9 ± 2.34
Ether extract	4.02 ± 0.25
**Pasteurized milk**	
Dry matter (%)	11.6 ± 0.47
Protein (%)	2.90 ± 0.15
Fat (%)	2.90 ± 0.37
Lactose (%)	4.82 ± 0.07
Somatic cell count	1093 ± 665
Bacteria (cfu)	271 ± 617
Milk urea nitrogen	12.0 ± 1.25

*Values are the mean ± standard deviation of nutrients from texturized calf starter and pasteurized waste milk. Bacterial counts were measured in colony forming units (cfu).*

### Donor Cows and Inocula Preparation

Primiparous Holstein cows (*n* = 4; 101.3 ± 5.3 days in milk; 37.8 ± 2.0 kg milk/d) provided a 60:40 forage to concentrate total mixed ration were housed in individual tie-stalls at the US Dairy Forage Research Center in Prairie du Sac, WI, United States. Cows were fed a total mixed ration containing corn and alfalfa silages, ground high moisture corn, protein byproduct supplements, and a vitamin/mineral mixture containing monensin once a day at 0700 h. On sampling and inoculation days, whole rumen contents (500 mL/cow) were collected from each donor at 0900 h. Bacteria-enriched (BE) inoculum was prepared by blending 250 mL of rumen contents from each cow (approximately 50% solid, 50% liquid) under CO_2_ with a Waring blender. The blended composite was strained through 4 layers of cheesecloth (44 × 36 threads per square in., Bleached/Grade 90) into two, 500 mL Thermo Scientific^TM^ Nalgene^TM^ PPCO Centrifuge Bottles. Rumen fluid and headspace was gassed with CO_2_ and centrifuged twice at 500 × *g* for 15 min at 25°C to ensure complete removal of feed particles and ciliate protozoa from the supernatant. The absence of ciliate protozoa was confirmed microscopically (Nikon LABOPHOT Biological Microscope, Tokyo, Japan) before oral administration, but do acknowledge the potential for low counts of protozoa to be present and not detectable by the limitations of microscopy. The pH and optical density at 600 nm were measured and the inoculum immediately stored at 39°C oven.

To prepare PE inocula, ruminal contents (225 mL/cow) were squeezed through four layers of cheesecloth, the rumen fluid was collected and composited rumen fluid was again filtered through four layers of cheesecloth. Rumen fluid was added to two, 1 L separatory funnels (400 mL to each) and an equal amount of sterile McDougall’s buffer warmed at 39°C was added to each funnel (McDougall, 1948). This was then gassed with CO_2_ and placed in a 39°C oven for 1 h. Ruminal contents containing the protozoal pellet (150 mL) were collected from the funnels into bottles maintained at 39°C and gassed with CO_2_. Protozoal activity was visualized microscopically before and after inoculation. Clarified rumen fluid used for the control group was prepared by collecting 300 mL supernatant from bacterial inoculum preparation. The supernatant was centrifuged at 12,000 × *g* at 4°C for 30 min to pellet rumen microbiota (Or-Rashid et al., 2007). The remaining supernatant was collected with a 60 cc catheter tip syringe and sterilized in an autoclave. During the experiment, PE and BE inocula were each prepared 10 times on the days of inoculation. BE and PE inocula samples (5 mL each) for VFA, NH_3_, bacterial community, and protozoal density analyses were collected immediately after preparation and frozen at −80°C.

### Rumen Inoculation and Sample Collection

Whole rumen contents (50 mL, average) were collected weekly by stomach intubation before ruminal dosing at 3–6 weeks of age. Calves were orally dosed once a week (4 total) with 50 mL treatment inocula which was followed by 50 mL 0.7% sterile saline (per veterinarian recommendation). Calves that received the PE and BE inocula were dosed with 50 mL of each type. A 145 cm long, 1.27 cm OD, PVC goat stomach tube was used to intubate calves at 3 and 4 weeks of age, while a 145 cm long, 1.59 cm OD, PVC medium equine stomach tube (Jorgensen Laboratories Inc., Loveland, CO, United States) for 5 and 6 weeks of age. The tubes did not contain strainers and allowed for the collection of fluids and solids. The tube was marked to denote the distance between the mouth to the 13th rib and was subsequently inserted through a 1.90 cm OD, 17.78 cm long, PVC speculum. A 400 mL oral drench syringe with the curved nozzle pipe removed (Labelvage, France) was used to withdraw rumen contents. Alternatively, the authors would recommend using a veterinary injection pump instead of the drench syringe for better collection ease (Nasco, Fort Atkinson, WI, United States). There were tubes dedicated to each inoculum type and they were washed between calves.

Rumen contents were immediately strained through 4 layers of cheesecloth; 5 mL collected with 5 mL of 50% formalin (v/v) for protozoal quantification stored at RT, 5 mL for DNA extraction, and 5 mL with 0.1 mL 50% H_2_SO_4_ stored at −80°C for VFA and NH_3_ analyses. Rumen fluid (4 mL) for VFA and NH_3_ analyses was thawed, mixed, centrifuged at 30,000 × *g* at 4°C for 30 min and the supernatant was collected and stored at −20°C. Ruminal VFA and NH_3_ were analyzed in-house using previously described gas-liquid chromatography and Lachat methods, respectively (Paula et al., 2018).

### Ruminal Ciliate Quantification

Visual quantification of ruminal ciliates were conducted as these methods are recognized as the gold standard for quantifying rumen ciliate protozoa (Dehority, 1984, 1993; Newbold et al., 2015). Briefly, 2 drops of brilliant green dye (2 g brilliant green and 2 mL glacial acetic acid diluted with 100 mL dH_2_0) were added to a 1 mL subsample of fixed ruminal fluid, vortexed, and allowed to remain at room temperature overnight. A drop of fixed rumen fluid from calves was placed on a microscope slide (5 slides/sample) with a coverslip to detect the presence or absence of protozoa at 100× and 400× magnification before proceeding to counting procedures. If all 5 slides did not contain ruminal protozoa, the animal was denoted as fauna-free. A subsample (0.25 mL) of the stained, fixed ruminal fluid sample was diluted with 14.5 mL of 30% glycerol (v/v) and a Sedgewick Rafter chamber (Structure Probe Inc., West Chester, PA, United States) with a 1 mm × 1 mm counting square was used to quantify protozoa. If the sample contained protozoa, a 1 mL of diluted sample was added to the chamber and counted twice at 100× magnification. Twenty-five squares were counted and the chamber was rotated 180° to count another 25 squares. Quantification (cell/ml) was calculated by the following:

$$\text{volume of 50 grids}=\text{1 mm}\times \text{1 mm}\times \text{1 mm (depth)}\times 50=50\text{mm}{}^{3}$$

volume of chamber=20⁢mm×50⁢mm×1⁢mm=1000mm=31mL

cells/mL=mean # of cells in 50 grids×1000/50×dilution

### DNA Extraction, Bacterial 16S rRNA Gene Amplification, and Sequencing

Rumen fluid (5 mL) was centrifuged at 10,000 × *g* for 30 min at 4°C to retain the microbial pellet. A total of 2 ml of lysis buffer (Yu and Morrison, 2004) was added to the pellet, mixed by vortexing, and 1 mL lysis buffer + pellet was added to a bead-beater tube. DNA was extracted following previously described methods (Yu and Morrison, 2004). DNA concentrations were quantified using a Qubit^®^ Fluorometer (Invitrogen, San Diego, CA, United States) with the Broad Range kit, and stored at −80°C. The V4 hypervariable region of the bacterial 16S rRNA gene was targeted with previously described universal primers (Kozich et al., 2013). Each PCR contained 50 ng DNA extract (ultrapure H_2_O for negative controls), 0.5 μL of forward and reverse primers, 6.5 μL ultrapure H_2_O, and 12.5 μL KAPA 2× HiFi HotStart ReadyMix (KAPA Biosystems Inc., Wilmington, MA, United States). Thermocycler conditions included denaturation at 95°C for 3 min with 25 cycles of 95°C for 30 s, annealing at 55°C for 30 s, 72°C for 30 s, and extension at 72°C for 30 s. PCR gel products (1% Molecular Biology Grade agarose, Gold Bio, Olivette, MO, United States) were cleaned by gel extraction with the ZR-96 Zymoclean Gel DNA kit (Zymo Research, Irvine, CA, United States). PCR products were quantified and pooled to equimolar concentrations to form the final DNA library for sequencing. The pooled library was sequenced with custom primers (Kozich et al., 2013) with a MiSeq 2 × 250 kit with 500 cycles (Illumina, San Diego, CA, United States) on an Illumina MiSeq at the US Dairy Forage Research Center (Madison, WI, United States). All raw DNA sequence reads were deposited in NCBI’s Sequence Read Archive under BioProject PRJNA454463.

### Bioinformatics Analyses

Demultiplexed samples were processed with MOTHUR v.1.39 using the online MiSeq SOP^2^ and the analyses described by Kozich et al. (2013). Paired-end sequence reads were combined and subsequently screened for low-quality reads (e.g., ambiguous bases, read length). Unique sequences were aligned with the silva.v4.fasta reference file and subsequently preclustered with 2 differences between sequences (i.e., 1 nucleotide difference for every 100 basepairs). UChime was used to identify chimeras that were subsequently removed (Edgar et al., 2011). Sequences were classified with the SILVA 16S Reference files and excluded taxa denoted as Archaea, Chloroplast, Cyanobacteria, Eukaryota, Mitochondria, and unknown. Uncorrected pairwise distances between sequences were calculated and sequences were clustered into operational taxonomic units (OTUs) with a 97% sequence similarity using the opticlust algorithm in MOTHUR. Good’s coverage, Inverse Simpson and Shannon Diversity indices, Chao1 richness and ACE richness were calculated. An analysis of similarity (ANOSIM) in MOTHUR was used to detect differences between bacterial community structures (Clarke, 1993). Samples were normalized to 6,000 sequences each. Beta-diversity was measured by calculating the Bray-Curtis (community structure) and Jaccard (composition) dissimilarity indices. A non-metric multidimensional scaling (nMDS) in R Studio (vegan package) was used to visualize rumen bacterial community structures and compositions.

### Blood Collection and Analyses

Blood (5 mL) was collected by jugular venipuncture within the first week of age for the measurement of total serum protein and detection of passive immunity transfer. Total protein was measured using an optical Brix refractometer. Whole blood was heparinized, kept on ice during sample collection, and centrifuged at 1,500 × *g* at 4°C for 15 min. Blood plasma (3 mL) was collected at 2, 4, 6, and 8 weeks of age and analyzed for glucose (Autokit Glucose, Wako Diagnostics, Mountain View, CA, United States) and plasma urea nitrogen (PUN; QuantiChrom Urea Assay Kit, BioAssay Systems, Hayward, CA, United States).

### Statistical Analyses

Inoculum type included calves inoculated with or without BE inoculum: BE(+) or BE(−), respectively, and with or without PE inoculum: PE(+) or PE(−), respectively. Means of inoculum type were evaluated with PROC MIXED in SAS (v 9.4, SAS Institute, Inc., Cary, NC, United States) with the random effect of calf and fixed effect of inoculum type. Differences by inoculum type were declared at *P* < 0.05 and trends at 0.05 ≤ *P* < 0.10. When multiple samples were collected over time, these time course data were evaluated as repeated measures with the PROC MIXED model in SAS from calf data. The model included calf as a random effect and PE, BE, and calf age, and the interaction of PE by calf age and BE by age as fixed effects. Differences by PE, BE, calf age and the interaction of PE by calf age and BE by age were declared at *P* < 0.05 and trends at 0.05 ≤ *P* ≤ 0.10. Means of PE and BE inocula were evaluated with PROC MIXED in SAS (v 9.4, SAS Institute, Inc., Cary, NC, United States) with the random effect of donor cow and fixed effect of microbial enrichment type.

## Results

### Rumen Inocula

Bacterial-enriched inocula were ciliate-free, while the PE inocula ranged from 9.8 × 10^4^ to 8.0 × 10^5^ and averaged 2.9 ± 2.9 × 10^5^ ciliates/mL rumen fluid. The pH and total VFA concentration of the PE inocula was greater, while the NH_3_ was 2-fold lower than the BE inocula (*P* < 0.001, Table 2). Individual molar proportions of VFA and diversity measurements did not differ by inoculum type. The bacterial community structure and composition differed by inocula type (ANOSIM, *P* < 0.001). Relative abundance of the phylum Bacteroidetes tended to be greater in the BE inocula than PE inocula (71.2 and 62.1 ± 2.1%, respectively, *P* = 0.10), whereas relative abundance of Firmicutes did not differ by inocula type (BE:19.2; PE: 20.2 ± 1.6%, *P* = 0.65). Relative abundance of the phylum Proteobacteria was greater in PE inocula than BE inocula (8.2 and 1.3 ± 1.0%, respectively, *P* < 0.001).

**TABLE 2:** Microbial inocula fermentation metabolites and diversity measures from primiparous lactating Holstein cows.

	**Bacteria-enriched inoculum**	**Protozoa-enriched inoculum**	**SE**	***P*-value**
**Rumen parameters**				
pH	5.83	7.14	0.07	<0.001
NH_3_ (mM)	6.84	3.67	0.50	<0.001
Total free amino acids (mM)	2.04	1.07	0.27	0.02
Total VFA (mM)	72.9	153	8.64	<0.001
Acetate (mol/100 mol)	59.7	58.4	0.57	0.13
Propionate	24.1	24.5	0.64	0.63
Butyrate	12.2	12.8	0.28	0.16
Isobutyrate	0.78	0.73	0.04	0.49
**Diversity measurements**				
Good’s coverage	94.6	93.9	0.00	0.11
Shannon Diversity index	4.99	5.13	0.09	0.27
Inverse Simpson index	52.3	55.1	7.28	0.79
ACE richness	1410	1686	136	0.17
CHAO richness estimator	1194	1318	86.24	0.32
# of OTUs	715	791	32	0.11
# of shared OTUs	313	317	n/a	n/a

*Rumen parameters and bacterial diversity measures are represented as the mean ± standard error (SE) from the bacterial (*n* = 10) and protozoal-enriched (*n* = 10) inocula administered to pre-weaned dairy calves. VFA, volatile fatty acids; OTUs, Operational taxonomic units.*

### Animal Performance

Starter DMI (0.55 ± 0.10 kg/d), ADG (0.95 ± 0.10 kg/d), body measurements, and PUN (22.2 ± 1.29 mg/dL) did not differ by treatment (Table 3). PUN was lower at 4 (19.4 mg/dL) and 6 (20.7 mg/dL) weeks of age than at 8 weeks (25.4 ± 1.49 mg/dL, *P* < 0.01; Table 3). Both starter DMI and BW increased with calf age (*P* < 0.001). Mean starter DMI was 0.11, 0.26, 0.42, 0.65 ± 0.08 kg/d at 3, 4, 5, and 6 weeks of age, respectively. Mean calf BW was 52.7, 58.5, 64.7, and 73.4 ± 1.7 kg at 3, 4, 5, and 6 weeks of age, respectively. Plasma glucose was greater in BE(+) calves than BE(−) calves (93.7 and 86.0 ± 2.24 mg/dL, respectively; *P* = 0.03) and differed by age (*P* < 0.001). Plasma glucose concentrations were greater at 2 (98.8 mg/dL), 4 (93.6 mg/dL), and 6 (92.7 mg/dL) than at 8 (73.2 ± 3.45 mg/dL) weeks of age. Calf fecal scores and rectal temperatures did not differ before and after inoculations. Mean nasal and eye scores were less than 1 and ear scores were 0 for all calves. Calves treated with the PE inoculum had lower rectal temperatures (38.4°C) than those not treated with PE inoculum (38.7 ± 0.1°C; *P* < 0.01). Fecal scores (1.42 ± 0.2) were not affected by main effect treatments; however, there was a significant interaction between the main effect treatments (*P* = 0.01) (Table 3).

**TABLE 3:** The main effects of microbial inoculum composition on calf performance, plasma metabolites, and ruminal environment.

	**Treatment**							
			
	**Bacteria-enriched (BE)**		**Protozoa-enriched (PE)**		**SE**	***P*-value**		
				
	**+**	**−**	**+**	**−**		**BE**	**PE**	**BE × PE**
**Animal performance**								
Dry matter intake (kg/d)	0.59	0.50	0.61	0.49	0.10	0.51	0.40	0.34
Average daily gain (kg/d)	0.99	0.90	1.02	0.88	0.07	0.37	0.17	0.13
Bodyweight (kg)	66.88	64.85	66.96	64.76	2.14	0.41	0.58	0.35
Body length (cm)	91.49	91.52	92.67	90.34	1.02	0.98	0.12	0.59
Paunch girth (cm)	101.74	100.45	101.14	101.04	1.53	0.56	0.96	0.06
Heart girth (cm)	94.13	94.03	94.45	93.71	1.03	0.95	0.62	0.29
Hip height (cm)	90.10	90.91	90.52	90.49	0.74	0.45	0.98	0.80
Hip width (mm)	192.68	189.34	191.80	190.22	2.54	0.36	0.67	0.89
Body temperature (°C)	38.55	38.63	38.44	38.74	0.07	0.42	< 0.01	0.13
Fecal score	1.33	1.52	1.44	1.42	0.17	0.36	0.92	0.01
**Blood plasma**								
Plasma urea N	21.92	22.42	21.72	22.63	1.29	0.78	0.62	0.71
Glucose	93.68	86.02	89.11	90.59	2.24	0.03	0.67	0.66
**Rumen parameters**								
NH_3_ (mM)	4.75	5.27	3.27	6.76	1.05	0.73	0.03	0.32
Total free amino acids (mM)	1.71	1.66	1.46	1.91	0.15	0.81	0.05	0.06
Total VFA (mM)	80.64	79.12	84.31	75.45	5.11	0.83	0.24	< 0.001
Acetate (mol/100 mol)	53.90	55.91	53.94	55.87	1.56	0.38	0.39	0.19
Propionate	31.07	32.35	32.82	30.60	1.25	0.48	0.22	0.37
Butyrate	10.81	8.32	9.72	9.43	0.78	0.04	0.79	0.33
Isobutyrate	0.45	0.48	0.32	0.60	0.09	0.81	0.05	0.18

*Animal performance, blood plasma metabolites, and rumen parameters are represented as the mean ± standard error (SE). A positive sign indicates a group of animals that received either the bacterial-enriched (BE, *n* = 10) or protozoal-enriched (PE, *n* = 10) inoculum whereas those with a negative sign did not receive either BE (*n* = 10) or PE (*n* = 10) inoculum.*

### The Main Effects of Inoculum Type on the Calf Rumen Microbial Ecosystem

Total VFA concentration did not differ by treatment, however, there was a treatment interaction between the main effects of BE and PE (*P* < 0.001). Calves treated with BE, PE, the combination of PE and BE, and clarified, autoclaved rumen fluid had 91.2, 99.2, 69.4, and 59.0 mM total ruminal VFA, respectively. No interaction between treatment and age was observed for individual VFA proportions or total VFA. The ruminal butyrate molar proportion was greater in BE(+) calves than BE(−) calves (10.8 and 8.32 ± 0.78%, respectively; *P* = 0.04), whereas molar proportions of acetate and propionate did not differ by treatment (Table 3). Ruminal NH_3_ concentration was lower in PE(+) calves than PE(−) calves (3.27 and 6.76 ± 1.05 mM, respectively, *P* = 0.04).

**FIGURE 1:**
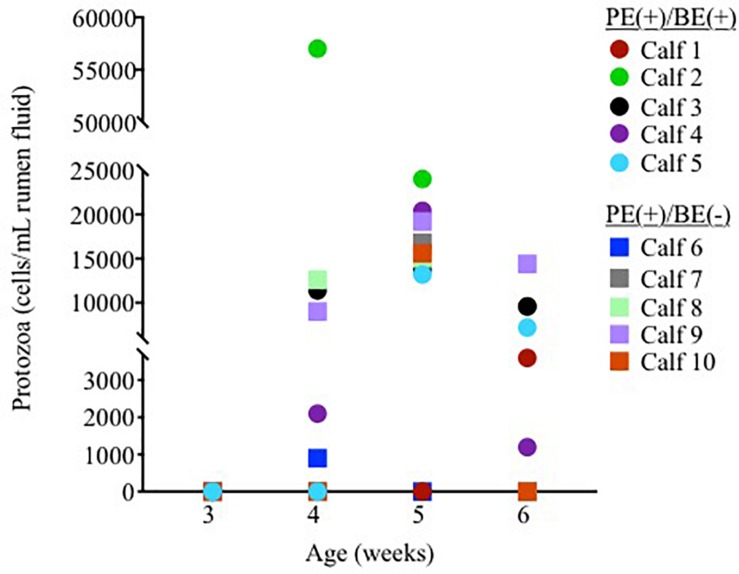
Individual rumen ciliate protozoal counts in pre-weaned dairy calves treated with the protozoal-enriched inoculum. Calves received either protozoa [PE(+)/BE(–)] or protozoa and bacteria-enriched [PE(+)/BE(+)] inocula.

Ciliate protozoa were observed in rumen fluid from 60, 80, and 60% of PE(+) calves (*n* = 10) at 4, 5, and 6 weeks of age, respectively (Figure 1). Ciliate counts averaged 1.5 × 10^4^/mL rumen fluid in these calves with protozoa belonging to the order Entodiniomorphida. The genus *Isotricha*, a member of the order Vestibuliferida, was found in the rumen fluid from Calf 2 at 4 weeks of age, but was not observed in all other calves or timepoints.

**FIGURE 2:**
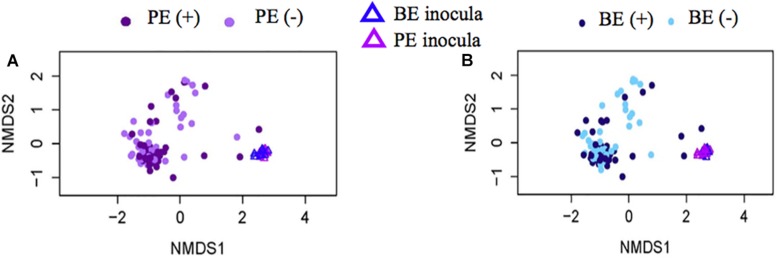
Non-metric multidimensional scaling (NMDS) plots of Bray-Curtis dissimilarity comparing rumen bacterial community structures between pre-weaned dairy calves and microbial inoculum. **(A)** PE (+) indicates a group of animals that received the protozoal-enriched (PE, dark purple circle) inoculum whereas PE (–) (light purple circle) indicates that the group did not receive the PE inoculum (stress = 0.82, RMSE = 5.1 × 10^–4^). **(B)** BE (+) indicates a group of animals that received the bacterial-enriched (BE, dark blue circle) inoculum whereas BE (–) (light blue circle) indicates that the group did not receive the BE inoculum (stress = 0.82, RMSE = 2.4 × 10^–4^). Purple and blue triangles represent the PE and BE inoculum prepared from the rumen contents from primiparous lactating cows, respectively.

**FIGURE 3:**
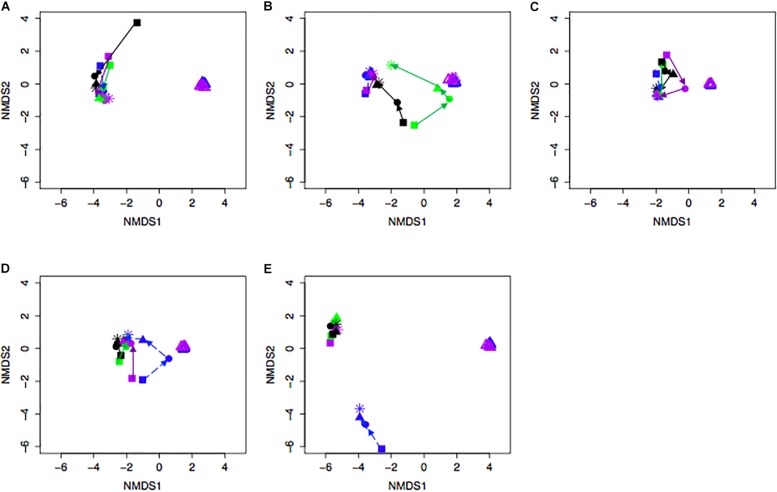
Non-metric multidimensional scaling (NMDS) plot of Bray-Curtis dissimilarity comparing rumen bacterial community structures between individual pre-weaned dairy calves and microbial inoculum. Purple and blue triangles represent the protozoal- (PE) and bacterial-enriched (BE) inocula prepared from the rumen contents from primiparous lactating cows, respectively. Age is indicated by shape: squares (week 3), circles (week 4), solid triangles (week 5), and asterisks (week 6). Each plot **(A–E)** depicts 4 different calves from 4 treatments. Treatments indicated by color: black [PE(–)/BE(–)], blue [PE(–)/BE(+)], purple [PE(+)/BE(–)], and green [PE(+)/BE(+)].

Bacterial community differences were observed between calves and the rumen inocula in the nMDS plots. An increase in the spread of Bray-Curtis values between calves and inocula was visualized (Figure 2). Rumen bacterial Shannon diversity index, inverse Simpson index, Good’s coverage, ACE richness, and number of OTUs did not differ by treatment, age, or treatment × age (Table 4). The rumen bacterial community structures did not differ by treatment, but were distinct from both the PE and BE inocula (ANOSIM, *P* < 0.001, Table 5). Two calves obtained similar rumen bacterial community structures to those of the inocula (Figure 3). One of these calves had a similar structure at 4 and 5 weeks of age, however, at 6 weeks of age the community was distinct from the inocula and similar to the other calves. This calf was inadvertently housed in a hutch that had a gap in the wire fence that enabled the calf to consume grass at week 4 of age. Once this gap was fixed at the end of week 4, the calf no longer had access to grass (Figure 3B, green). The other calf was dosed with the BE inoculum and had a similar community at 4 weeks of age (Figure 3D, blue).

**TABLE 4:** The main effects of microbial inoculum composition on pre-weaned dairy calf rumen bacterial diversity.

	**Treatment**							
			
	**Bacteria-enriched (BE)**		**Protozoa-enriched (PE)**		**SE**	***P*-value**		
				
	**+**	**−**	**+**	**−**		**BE**	**PE**	**BE × PE**
Good’s coverage	99.1	99.2	99.1	99.2	0.00	0.55	0.47	0.34
Shannon diversity index	2.82	2.78	2.84	2.76	0.13	0.86	0.65	0.84
Inverse simpson index	11.1	8.73	10.7	9.11	1.88	0.39	0.56	0.84
ACE richness	262	232	270	224	32.0	0.52	0.32	0.31
CHAO richness estimator	225	203	228	201	28.6	0.59	0.51	0.28
# of OTUs	148	133	145	135	18.9	0.57	0.64	0.39

*Diversity measures are represented as the mean ± standard error (SE). A positive sign indicates a group of animals that received either the bacterial-enriched (BE, *n* = 10) or protozoal-enriched (PE, *n* = 10) inoculum whereas those with a negative sign did not receive either BE (*n* = 10) or PE (*n* = 10) inoculum.*

**TABLE 5:** Analyses of molecular variance and similarities of the rumen bacterial communities from pre-weaned dairy calves and microbial inocula.

	**AMOVA**	**ANOSIM**	
		
**Comparison**	***P*-value**	***R*-value**	***P*-value**
week 3-week 4	0.07	0.06	0.05
week 3-week 5	< 0.001	0.17	< 0.001
week 3-week 6	< 0.001	0.28	< 0.001
week 4-week 5	0.86	–0.02	0.76
week 4-week 6	0.12	0.06	0.05
week 5-week 6	0.40	0.026	0.18
PE (+)-PE (−)	0.07	–0.06	0.56
BE (+)-BE (−)	0.41	7.67 × 10^–4^	0.42
BE (+)-BE inoculum	< 0.001	0.68	< 0.001
BE (−)-BE inoculum	< 0.001	0.67	< 0.001
PE (+)-PE inoculum	< 0.001	0.46	< 0.001
PE (−)-PE inoculum	< 0.001	0.38	< 0.001
BE inoculum-PE inoculum	< 0.001	0.63	< 0.001

*Analysis of molecular variance (AMOVA) and analysis of similarity (ANOSIM) were used to draw comparisons by calf age (3–6 weeks), within treatment groups, and between treatment groups and the two inocula sources, and between inocula sources. Bacterial communities were dissimilar if *P* < 0.05.*

**FIGURE 4:**
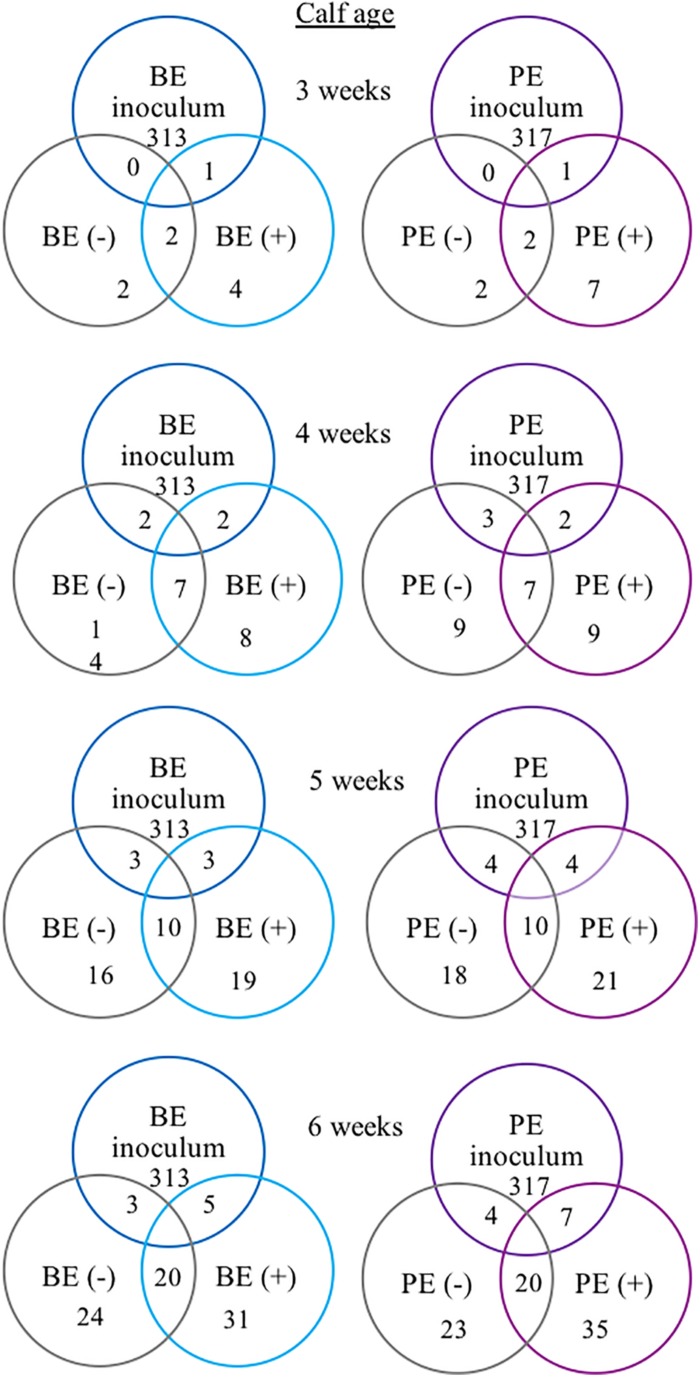
Number of shared bacterial operational taxonomic units (OTUs) between rumen inocula and pre-weaned dairy calves 3–6 weeks of age. The values represent the number of shared OTUs between and within groups. Comparisons are shown within and between treatment groups and the inoculum source. A positive sign indicates a group of animals that received either the bacterial-enriched (BE) or protozoal-enriched (PE) inoculum whereas those with a negative sign did not.

The following 9 OTUs were related to the taxa listed in parentheses: OTU 1 (unclassified *Prevotella*), OTU 3 (unclassified *Prevotella*), OTU 5 (unclassified Gammaproteobacteria), OTU 7 (Erysipelotrichaceae), OTU 9 (unclassified *Prevotella*), OTU 48 (unclassified *Prevotella*), OTU 49 (Veillonellaceae), OTU 109 (Lachnospiraceae), and OTU 118 (unclassified Bacteria). The number of shared OTUs amongst all calves at 3, 4, 5, and 6 weeks of age was 2, 7, 10, and 20 OTUs, respectively. PE(+) and BE(+) calves shared 1 OTU (#49), whereas the PE(−) and BE(−) calves did not share any OTUs with the PE and BE inocula at 3 weeks of age (Figure 4). PE(+) calves and the PE inoculum shared 7 OTUs (#1, 3, 5, 7, 48, 109, 118) and the PE (−) calves shared 4 OTUs (#1, 5, 7, 118) with the PE inoculum at 6 weeks of age. BE(+) calves and the BE inoculum shared 5 OTUs (#1, 3, 5, 9, 118), and the BE (−) calves shared 3 OTUs (#1, 5, 118) with the BE inoculum at 6 weeks of age. Calves treated with the PE inoculum shared 7, 9, 21, and 35 total OTUs, while those treated with the BE inoculum shared 4, 8, 19, and 35 total OTUs at 3–6 weeks of age, respectively (Figure 4). The number of OTUs at 3,4,5 and 6 weeks of age was 139, 167, 138, and 118 ± 27 and did not differ by calf age.

**FIGURE 5:**
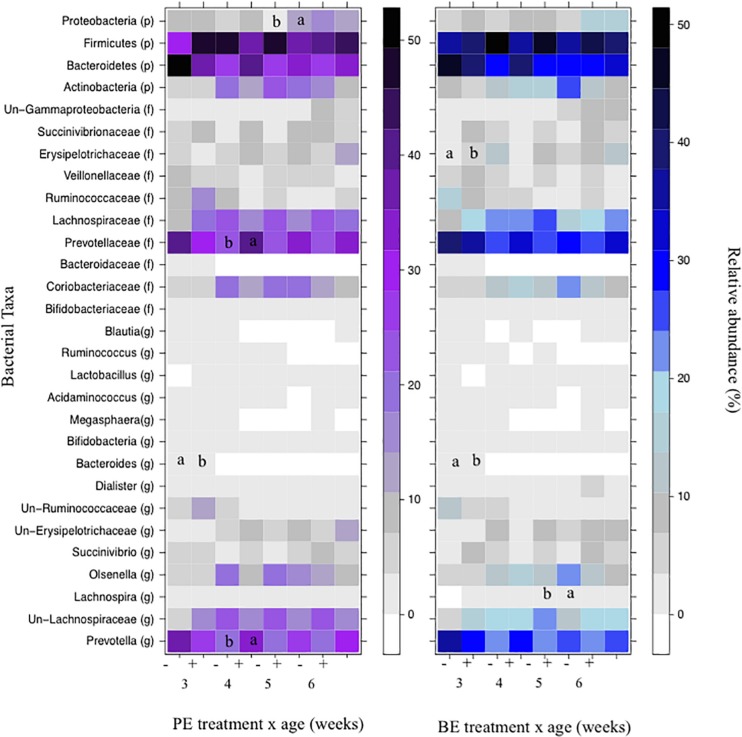
The effect of treatment by calf age on the relative abundance of rumen bacterial taxa from pre-weaned dairy calves. A positive sign indicates a group of animals that received either the bacterial-enriched (BE) or protozoal-enriched (PE) inoculum whereas those with a negative sign did not receive the BE or PE inoculum. Numbers on the *x*-axis 3–6 represent calf age. Colored squares represent the mean relative abundance of a given bacterial taxa, phylum (p), family (f), and genus (g). Un refers to unclassified taxa. Mean relative (%) abundances of taxa without a common letter a or b differ (*P* < 0.05).

The relative abundances of bacterial taxa (i.e., phyla, family, and genus) did not differ by the main effects of PE and BE (Supplementary Table 1). Bacteria belonging to the Bacteroidetes and Firmicutes phyla, Lachnospiraceae family, and *Prevotella* genus were affected by the interaction of PE inoculum and age (*P* < 0.05) (Figure 5). The relative abundance of Firmicutes was quadratically increased by the interactions of BE × age (*P* = 0.04) and PE × age (*P* < 0.01). The relative abundance of *Prevotella* tended to be quadratically (*P* = 0.06) and cubically (*P* = 0.06) increased by the interaction of PE × age. PE(+) calves had greater relative abundances of the genus *Prevotella* (33.0 vs. 18.0 ± 5.3%) and phylum Proteobacteria (12.4 vs. 2.3 ± 3.2%) at 4 and 5 weeks of age than PE(−) calves, respectively (Figure 5).

### The Effect of Calf Age on the Rumen Microbial Ecosystem

Total amino acids and butyrate molar proportions did not differ by age. Ruminal molar proportions of acetate linearly decreased, whereas propionate and total VFA linearly increased with age (*P* < 0.001) (Table 6). Ruminal NH_3_ was lower at 6 weeks (2.72 mM) than 3–5 weeks of age (6.00, 4.86, and 4.51 ± 0.85 nM), respectively (*P* = 0.02).

**TABLE 6:** The effect of dairy calf age on rumen bacterial taxa and rumen parameters.

	**Age (weeks)**				***P*-value**			
		
	**3**	**4**	**5**	**6**	**SE**	**Linear**	**Quadratic**	**Cubic**
**Bacterial taxa**								
Bacteroidetes (p)	43.1	34.2	30.0	30.5	3.66	0.02	0.12	0.99
Firmicutes (p)	39.0	41.7	40.9	41.3	4.07	0.74	0.71	0.70
Proteobacteria (p)	7.76	6.88	7.36	15.2	2.29	0.04	0.02	0.40
Actinobacteria (p)	6.63	14.9	19.9	10.9	3.29	0.24	< 0.01	0.25
Un-Gammaproteobacteria (f)	1.44	2.17	2.94	8.25	1.25	< 0.01	0.05	0.34
Succinivibrionaceae (f)	5.26	4.18	3.98	6.50	2.03	0.70	0.24	0.75
Erysipelotrichaceae (f)	3.36	7.14	6.83	8.94	1.55	0.03	0.53	0.20
Veillonellaceae (f)	7.13	3.39	4.23	3.76	1.12	0.08	0.14	0.22
Ruminococcaceae (f)	11.3	5.54	3.39	4.16	1.70	< 0.01	0.07	0.93
Lachnospiraceae (f)	13.4	21.2	20.2	21.1	3.43	0.15	0.18	0.25
Prevotellaceae (f)	36.6	30.4	27.2	28.8	3.78	0.13	0.21	0.88
Bacteroidaceae (f)	1.14	0.03	0.01	0.02	0.27	< 0.01	0.05	0.39
Coriobacteriaceae (f)	5.46	14.1	18.2	10.4	3.19	0.19	< 0.01	0.41
Bifidobacteriaceae (f)	1.06	0.75	1.71	0.45	0.38	0.63	0.21	0.04
*Ruminococcus* (g)	0.58	0.23	0.14	0.02	0.09	< 0.001	0.22	0.48
*Lactobacillus* (g)	0.09	0.15	0.33	0.24	0.07	0.06	0.29	0.20
*Acidaminococcus* (g)	1.37	1.31	0.17	0.25	0.58	0.09	0.91	0.39
*Megasphaera* (g)	1.38	0.06	0.03	0.21	0.19	< 0.001	< 0.001	0.24
*Bifidobacteria* (g)	1.06	0.75	1.71	0.45	0.38	0.63	0.21	0.04
*Bacteroides* (g)	1.14	0.03	0.01	0.02	0.27	< 0.01	0.05	0.39
Un-Gammaproteobacteria (g)	1.44	2.17	2.94	8.25	1.25	< 0.01	0.05	0.34
Un-Ruminococcaceae (g)	8.79	2.91	0.93	0.43	1.60	< 0.01	0.09	0.70
Un-Erysipelotrichaceae (g)	2.84	6.23	6.08	8.55	1.55	0.05	0.76	0.29
Un-Prevotellaceae (g)	4.29	3.87	3.16	3.87	0.96	0.61	0.55	0.71
*Succinivibrio* (g)	5.27	4.18	3.98	6.50	2.03	0.70	0.24	0.75
*Olsenella* (g)	5.46	14.1	18.2	10.4	3.19	0.19	< 0.01	0.41
*Lachnospira* (g)	0.14	0.16	0.23	0.10	0.04	0.83	0.04	0.06
Un-Lachnospiraceae (g)	13.4	21.2	20.2	21.1	3.43	0.15	0.18	0.25
*Prevotella* (g)	32.2	26.0	23.9	24.9	3.73	0.17	0.23	0.94
*Dialister* (g)	1.15	0.87	1.37	2.52	0.50	0.05	0.15	0.95
**Rumen volatile fatty acids**								
Total VFA (mM)	66.7	75.6	87.6	89.6	5.88	< 0.01	0.53	0.57
Acetate (mol/100 mol)	60.5	55.5	52.2	51.5	1.57	< 0.001	0.09	0.83
Propionate	26.4	30.8	33.9	35.7	1.44	< 0.001	0.32	0.97
Butyrate	9.59	9.67	9.97	9.06	0.91	0.76	0.55	0.66
Isobutyrate	0.57	0.53	0.48	0.27	0.09	0.03	0.23	0.50

*The values are the mean relative % abundance ± standard error (SE) of the most abundant bacterial taxa: p (phylum), f (family), and g (genus) and mean ± SE of the rumen volatile fatty acids (VFA). Un refers to taxa that are unclassified at the family or genus level.*

Bacterial communities at 3 weeks differed from those at 5 and 6 weeks of age (AMOVA and ANOSIM, *P* < 0.001), however, no differences in community structure were observed between 4, 5, and 6 weeks of age. Relative abundance of the bacterial phyla Firmicutes did not differ by age. Proteobacteria linearly and quadratically increased, Bacteroidetes linearly decreased, and Actinobacteria quadratically increased by age (*P* < 0.05) (Table 6). Relative abundances of bacteria belonging to the Ruminococcaceae and Bacteroidaceae families linearly decreased with age. Relative abundances of Bifidobacteriaceae, Lachnospiraceae, Prevotellaceae, and Veillonellaceae did not differ by age. The relative abundances of the genera, *Bacteroides*, *Megasphaera*, and *Ruminococcus* linearly decreased by age (*P* < 0.01) (Table 6). Calf age did not affect the relative abundances of *Bifidobacteria*, *Prevotella*, and *Succinivibrio* (Table 6).

## Discussion

The most opportune period to direct the microbial ecology of a ruminant may occur during ruminal development and prior to natural microbial establishment (Yáñez-Ruiz et al., 2015). Rumen-derived inocula and probiotics were previously used to manipulate the ruminal environment and influence animal performance. These included rumen boluses and fluid from adult ruminants. Several microbial domains are present in these inocula, yet their impact on the developing ruminal microbial environment and calf health and growth is not well described. The present study focused on the effect of ruminal bacterial- and protozoal-enriched inocula on rumen bacterial ecology and performance of dairy calves.

Dairy calves are functionally non-ruminants for approximately the first 3 weeks of age until they start to increase their solid feed intake between 3 and 8 weeks in age, resulting in increased microbial fermentation (Baldwin et al., 2004; Dias et al., 2017). Dairy calves offered concentrate in addition to milk produced more VFA in comparison to those offered only milk (Dias et al., 2017). In the present study, an increase in starter DMI with calf age was simultaneously observed with an increase in total VFA regardless of inocula type. An interaction between the main effects BE × PE inocula on total VFA demonstrated the potential for either microbial inocula to contribute to altered microbial fermentation.

Butyrate is the main energy source for epithelial cells, promotes the development of rumen papillae, and accelerates the development of the rumen (Baldwin et al., 2004; Wang, 2012; Song et al., 2018). The lactate-utilizing bacteria, including, *Megasphaera elsdenii*, convert lactic acid to butyrate and increased ruminal butyrate when orally dosed into pre-weaned calves (Muya et al., 2015). The more abundant bacterial families Lachnospiraceae and Ruminococcaceae are also able to produce butyrate through the breakdown of complex polysaccharides, including starch (Vital et al., 2014). Relative abundances of *Megasphaera*, and the families Lachnospiraceae and Ruminococcaceae did not differ between BE(+) and BE(−) calves, but molar proportions of butyrate were greater in BE(+) than BE(−) calves suggesting an alternative contribution of the BE inoculum to butyrate concentration. The relative abundance of Ruminococcaceae and *Megasphaera* linearly decreased by age. However, the lack of change with age in the more abundant butyrate-producer, Lachnospiraceae, likely contributed to no change in ruminal butyrate concentration with age.

Little to no changes in the rumen bacterial communities were observed between calves dosed with or without BE inoculum and with or without PE inoculum. Calf rumen bacterial communities from the present study were more similar to each other than to the BE and PE inocula. Rumen bacterial communities were dissimilar between 8 week-old dairy calves and lactating cows and similar between 1 and 2 year old cows (Dill-McFarland et al., 2017). Two calves had rumen bacterial communities similar to the inocula at individual time points during the study. One of these calves was treated with both the PE and BE inocula (Calf 2, Figure 1) and the other with only the BE inoculum. Although the calf bacterial community structure from these 2 calves transiently shifted toward that of the adult lactating cow donor without fully changing to that structure, further studies should be performed to determine if and how these changes can be sustained.

In agreement with previous studies, the rumen bacterial community structure and composition did change with calf age (Rey et al., 2014; Dias et al., 2017; Dill-McFarland et al., 2017). Direct-fed microbial supplementation did not impact bacterial alpha diversity measures in pre- or post-weaned calves and bacterial communities clustered by age rather than by treatment (Fomenky et al., 2018). When bacteria naturally established in the rumen the communities clustered by age, however, the Shannon diversity index did not change (Rey et al., 2014). Several factors including diet, weaning age, housing, and host genetics may contribute to the changes in the bacterial communities over time (Khan et al., 2016; Meale et al., 2016; Dias et al., 2017). The transition from milk to solid-based diets alters the rumen microbial community as solid feed enters the rumen instead of bypassing the rumen to the abomasum and provides substrates for microbial fermentation (Abe et al., 1979; Church, 1988). These shifts were observed to occur between 3 and 5 weeks of age as starter DMI increased through 6 weeks of age, while total ruminal VFA concentrations and propionate proportions linearly increased.

Relative abundances of bacterial taxa changed by calf age and the interaction of inocula treatment x age (Figure 3). The treatment x age interaction demonstrates a potential challenge to effectively alter the ruminal environment in a growing ruminant with a donor-generated inoculum. Consistent with this observation is that age was a more significant factor than inoculum treatment in lambs dosed with fibrolytic bacterial cultures generated from moose rumen fluid (Ishaq et al., 2015). In contrast, more consistent alterations to specific gut bacterial taxa and calf performance have been observed through the oral dosing of pure bacterial cultures recognized as probiotics (Lkováa et al., 2010; Muya et al., 2015; Rodriguez-Palacios et al., 2017).

In agreement with previous studies, the most abundant bacterial phyla included Bacteroidetes, Firmicutes, Proteobacteria, and Actinobacteria (Rey et al., 2014; Meale et al., 2016). Relative abundances of Proteobacteria between PE(+) and PE(−) treated calves did not differ despite a greater abundance of this phyla in the PE than BE inoculum. Relative abundance of Firmicutes remained stable, while Bacteroidetes declined between 3 and 6 weeks of age. Relative abundances of the genus *Prevotella* did not change with age as was also observed in bull calves between 14 and 42 days of age (Li et al., 2012). It is unclear why PE(+) treated calves had a 15.4% increase in relative abundance of *Prevotella* only at 4 weeks of age in comparison to PE(−) calves in this study. The fibrolytic specialists *Lachnospira* and *Ruminococcus* did fluctuate with calf age, but were less abundant in comparison to calves fed a partial hay diet that would provide more substrate for bacterial adherence (Biddle et al., 2013; Rey et al., 2014).

Direct-fed microbials and probiotic supplements positively impact calf growth and health, yet little information is known about the effects of rumen-derived inocula on these parameters (Rodriguez-Palacios et al., 2017; Fomenky et al., 2018; Sharma et al., 2018). The relative abundance of the genera *Lactobacillus* and *Bifidobacteria*, frequently used probiotics, did not differ by inoculum type or calf age. Calf performance, measured as feed-conversion ratio and ADG, was not different between faunated and defaunated calves in a prior inoculation study (Schönhusen et al., 2003). BE and PE inocula were safely administered to dairy calves and did not impact calf health and growth measurements after 4 total inoculations. Preweaned calves are pseudo-monogastrics that mainly rely on digestive enzymes to breakdown milk instead of rumen microbiota (Longenbach and Heinrichs, 1998). This physiological characteristic may be why changes in growth were not observed between treatments, despite changes in fermentation parameters.

There is strong causal evidence that the presence of ruminal ciliate protozoa results in an increase in ruminal NH_3_ concentration and is considered to be due to greater bacterial protein breakdown by the protozoa and/or fermentable substrate sequestration (Veira et al., 1983; Yáñez-Ruiz et al., 2007; Newbold et al., 2015; Abecia et al., 2017). In contrast to previous observations, in this study, PE (+) calves subsequently faunated with ruminal ciliate protozoa had lower concentrations of rumen NH_3_, free AA, and isobutyrate which are released on the breakdown of protein. The reason for the difference from other literature observations is not known, but it could be related to the stage of the ruminal microbial community development, substrate type, ruminal pH or some other unidentified factor.

Few studies have focused on the effect of faunation on pre-weaned dairy calves. The studies that have been conducted with calves provided access to forage and less concentrate (Pounden and Hibbs, 1950; Eadie, 1962b; Schönhusen et al., 2003) in comparison to our present study which was conducted using a feed ration higher in fermentable carbohydrates comparable to current dairy industry practices. Calves dosed with ruminal fluid had an increase in protozoa with age and an increase in NH_3_ in comparison to those not dosed (Schönhusen et al., 2003), yet calves from this study consumed a mixed forage/concentrate diet. Calves inoculated with ciliates at 8 days of age had a ciliate population by 15 days of age, but when calves were weaned on 100% concentrate diets ciliates disappeared, while a an equal mixture of forage:concentrate maintained a ruminal ciliate population (Eadie, 1962b). Although calves from the present study were inoculated with ciliates once a week, the solid portion of the diet was 100% calf starter, unlike the donor cows that consumed a 60:40 forage:concentrate diet. The one calf that had uncontrolled access to, and was observed to consume, cool-season grasses ate less starter (14 g DM/d vs. 338 g DM/d) at 4 weeks of age. This calf also had a mixed ciliate population until the fence was fixed. Lambs with a ruminal pH less than 6.0 had small entodinia present, while those with a ruminal pH greater than 6.5 had a mixed ciliate population (Eadie, 1962b). As the calves from this study were on a mixed whole milk and concentrate diet with a starch content of 39%, it is suspected that their ruminal pH was less than 6.5 during this time as has been observed in other studies (Suarez-Mena et al., 2015).

## Conclusion

The results of the present study demonstrate the challenges of directing the developing rumen microbial environment and bacterial community, while showing changes in the ruminal environment with age. The administration of ruminal inocula did not alter measured parameters of calf health and growth. Ruminal fermentation by-products were altered by microbial inoculum type, despite no changes in bacterial diversity and few differences in bacterial taxa. Butyrate proportions were greater in BE (+) pre-weaned calves, future research to determine if this impacted ruminal papillae growth and other measurements of gut health (e.g., immune function) are suggested. The presence of a ruminal ciliate population in PE (+) treated calves was not maintained with calf age and may have been due to the preweaned calf diet and ruminal environment. However, the ability to direct the ruminal bacterial community structure of pre-weaned calves toward that of the adult donors was temporarily successful in two calves.

## Data Availability

The datasets generated for this study can be found in NCBI Sequence Read Archive, PRJNA454463.

## Ethics Statement

This study was carried out in accordance with the recommendations of the University of Wisconsin’s Institutional Animal Care and Use Committee. The protocol was approved by the University of Wisconsin’s Institutional Animal Care and Use Committee under the protocol A005829.

## Author Contributions

LC and GZ developed the study design and prepared the ruminal inocula. All authors took calf performance measurements, performed blood draws, collected rumen fluid samples, and administered the inocula. LC performed molecular biology techniques, performed bioinformatics analyses, prepared and counted ruminal ciliates, and prepared the manuscript. WR analyzed ruminal VFA and ammonia. GZ and WR reviewed and edited the manuscript. All authors read and approved the final version of the manuscript.

## Conflict of Interest Statement

The authors declare that the research was conducted in the absence of any commercial or financial relationships that could be construed as a potential conflict of interest.

## Footnotes

1 https://www.vetmed.wisc.edu/dms/fapm/fapmtools/8calf/calf_health_scoring_chart.pdf (accessed February 5, 2019).

2 https://www.mothur.org/wiki/MiSeq_SOP, visited 5/11/19.
