# Supplementary material for: Microbial Inoculum Composition and Pre-weaned Dairy Calf Age Alter the Developing Rumen Microbial Environment
Source: Front Microbiol. 2019 Jul 23;10:1651. doi: 10.3389/fmicb.2019.01651 (PMC6664089; doi:10.3389/fmicb.2019.01651)
Supplement: Supplementary file 1 [file Table_1.DOCX]

Supplementary Table 1| The effect of treatment on the relative abundance of rumen bacterial taxa from pre-weaned dairy calves. A positive sign indicates a group of animals that received either the bacterial-enriched (BE) or protozoal-enriched (PE) inoculum whereas those with a negative sign did not receive the BE or PE inoculum. Bacterial taxa include phylum (p), family (f), and genus (g). Un refers to unclassified taxa.

|  | Treatment | | | |  |  | | |
| --- | --- | --- | --- | --- | --- | --- | --- | --- |
|  | Bacteria-enriched (BE) | | Protozoa-  enriched (PE) | | SE | *P*-value | | |
| **Bacterial Taxa** | + | - | + | - |  | BE | PE | BE x PE |
| Bacteroidetes (p) | 35.5 | 33.4 | 36.4 | 32.5 | 3.54 | 0.68 | 0.45 | 0.46 |
| Firmicutes (p) | 38.4 | 43.0 | 40.1 | 41.4 | 4.07 | 0.43 | 0.83 | 0.56 |
| Proteobacteria (p) | 9.46 | 9.13 | 10.8 | 7.76 | 2.16 | 0.92 | 0.32 | 0.80 |
| Actinobacteria (p) | 14.5 | 11.7 | 10.7 | 15.4 | 3.27 | 0.55 | 0.32 | 0.68 |
| Un-Gammaproteobacteria (f) | 4.58 | 2.82 | 3.69 | 3.70 | 1.07 | 0.26 | 0.99 | 0.49 |
| Succinivibrionaceae (f) | 4.32 | 5.65 | 6.56 | 3.41 | 2.06 | 0.65 | 0.30 | 0.86 |
| Erysipelotrichaceae (f) | 5.68 | 7.45 | 8.14 | 4.99 | 1.43 | 0.39 | 0.13 | 0.02 |
| Veillonellaceae (f) | 3.87 | 5.38 | 4.03 | 5.22 | 0.82 | 0.21 | 0.32 | 0.95 |
| Ruminococcaceae (f) | 4.74 | 7.43 | 6.23 | 5.95 | 1.16 | 0.12 | 0.87 | 0.49 |
| Lachnospiraceae (f) | 19.2 | 18.8 | 17.7 | 20.3 | 3.52 | 0.94 | 0.60 | 0.46 |
| Prevotellaceae (f) | 31.8 | 29.7 | 33.7 | 27.8 | 3.58 | 0.68 | 0.25 | 0.57 |
| Bacteroidaceae (f) | 0.04 | 0.56 | 0.08 | 0.52 | 0.18 | 0.07 | 0.11 | 0.12 |
| Coriobacteriaceae (f) | 13.4 | 10.6 | 9.72 | 14.3 | 3.24 | 0.55 | 0.32 | 0.69 |
| Bifidobacteriaceae (f) | 1.03 | 0.96 | 0.94 | 1.04 | 0.29 | 0.87 | 0.81 | 0.81 |
| *Ruminococcus* (g) | 0.16 | 0.32 | 0.20 | 0.29 | 0.07 | 0.13 | 0.35 | 0.02 |
| *Lactobacillus* (g) | 0.25 | 0.15 | 0.20 | 0.21 | 0.06 | 0.27 | 0.92 | 0.65 |
| *Acidaminococcus* (g) | 0.52 | 1.03 | 0.37 | 1.18 | 0.42 | 0.40 | 0.20 | 0.60 |
| *Megasphaera*(g) | 0.39 | 0.45 | 0.37 | 0.46 | 0.13 | 0.74 | 0.61 | 0.27 |
| *Bifidobacteria* (g) | 1.03 | 0.96 | 0.94 | 1.04 | 0.29 | 0.87 | 0.81 | 0.81 |
| *Bacteroides* (g) | 0.04 | 0.56 | 0.08 | 0.52 | 0.18 | 0.07 | 0.11 | 0.12 |
| Un-Gammaproteobacteria (g) | 4.58 | 2.82 | 3.69 | 3.70 | 1.07 | 0.26 | 0.99 | 0.49 |
| Un-Ruminococcaceae (g) | 1.98 | 4.55 | 3.64 | 2.90 | 1.26 | 0.17 | 0.68 | 0.86 |
| Un-Erysipelotrichaceae (g) | 5.04 | 6.81 | 7.29 | 4.56 | 1.17 | 0.31 | 0.12 | 0.01 |
| Un-Prevotellaceae (g) | 4.07 | 3.53 | 3.94 | 3.66 | 0.72 | 0.60 | 0.79 | 0.93 |
| *Succinivibrio* (g) | 4.31 | 5.65 | 6.55 | 3.41 | 2.06 | 0.65 | 0.30 | 0.86 |
| *Olsenella* (g) | 13.4 | 10.7 | 9.73 | 14.3 | 3.24 | 0.55 | 0.32 | 0.69 |
| *Lachnospira* (g) | 0.22 | 0.09 | 0.16 | 0.16 | 0.04 | 0.06 | 1.00 | 0.44 |
| Un-Lachnospiraceae (g) | 16.6 | 16.9 | 15.3 | 18.1 | 3.46 | 0.95 | 0.57 | 0.52 |
| *Prevotella* (g) | 27.4 | 26.1 | 29.6 | 23.8 | 3.64 | 0.79 | 0.27 | 0.58 |
| *Dialister* (g) | 1.21 | 1.74 | 1.19 | 1.76 | 0.39 | 0.36 | 0.32 | 0.68 |
